# Comparing laparoscopic antireflux surgery with esomeprazole in the management of patients with chronic gastro-oesophageal reflux disease: a 3-year interim analysis of the LOTUS trial

**DOI:** 10.1136/gut.2008.148833

**Published:** 2008-05-09

**Authors:** L Lundell, S Attwood, C Ell, R Fiocca, J-P Galmiche, J Hatlebakk, T Lind, O Junghard, on behalf of the LOTUS trial collaborators

**Affiliations:** 1 Department of Surgery, Karolinska University Hospital, Huddinge, Sweden; 2 Department of Surgery, North Tyneside General Hospital, North Shields, Tyne and Wear, UK; 3 Department of Gastroenterology, Dr Horst Schmidt-Hospital, Wiesbaden, Germany; 4 Department of Surgical and Morphological Sciences, Anatomic Pathology Division, University of Genova, Italy; 5 Department of Gastroenterology and Hepatology, Nantes University and CIC INSERM, Nantes, France; 6 Institute of Medicine, Haukeland University Hospital, University of Bergen, Norway; 7 Astra Zeneca R & D, Mölndal, Sweden

## Abstract

**Background:**

With the introduction of laparoscopic antireflux surgery (LARS) for gastro-oesophageal reflux disease (GORD) along with the increasing efficacy of modern medical treatment, a direct comparison is warranted. The 3-year interim results of a randomised study comparing both the efficacy and safety of LARS and esomeprazole (ESO) are reported.

**Methods:**

LOTUS is an open, parallel-group multicentre, randomised and controlled trial conducted in dedicated centres in 11 European countries. LARS was completed according to a standardised protocol, comprising a total fundoplication and a crural repair. Medical treatment comprised ESO 20 mg once daily, which could be increased stepwise to 40 mg once daily and then 20 mg twice daily in the case of incomplete GORD control. The primary outcome variable was time to treatment failure (Kaplan–Meier analysis). Treatment failure was defined on the basis of symptomatic relapse requiring treatment beyond that stated in the protocol.

**Results:**

554 patients were randomised, of whom 288 were allocated to LARS and 266 to ESO. The two study arms were well matched. The proportions of patients who remained in remission after 3 years were similar for the two therapies: 90% of surgical patients compared with 93% medically treated for the intention to treat population, p = 0.25 (90% vs 95% per protocol). No major unexpected postoperative complications were experienced and ESO was well tolerated. However, postfundoplication complaints remain a problem after LARS.

**Conclusions:**

Over the first 3 years of this long-term study, both laparoscopic total fundoplication and continuous ESO treatment were similarly effective and well-tolerated therapeutic strategies for providing effective control of GORD.

During recent years, there has been some debate as to the relative value of long-term proton pump inhibitor (PPI) treatment compared with antireflux surgery for the management of chronic gastro-oesophageal reflux disease (GORD). The suboptimal level of health-related quality of life in patients with GORD illustrates the importance of prompt and aggressive treatment when the disease manifestations are not fully under control.^1^^–^3

With the introduction by Nissen of the fundoplication procedure,^4^ this operation has been found to be effective and widely used throughout different parts of the world, although there are concerns relating to the safety of the procedure per se, and the mechanical side effects and durability of the antireflux repair in particular.^5^^–^8 Although the perioperative and postoperative courses have been facilitated by the introduction of the laparoscopic technology,^9^ the results in community practice remain far from optimal, and data on the long-term efficacy of standardised laparoscopic antireflux surgery (LARS) are lacking.^6^ ^7^ The poor therapeutic results in community practice may be due to variability in procedures or lack of experience of the surgeons, so there is a need to standardise and monitor the surgical procedures.

In a recently published study,^10^ open antireflux surgery and medical treatment in the form of daily omeprazole treatment were compared in patients with reflux oesophagitis. After 7 years of follow-up, more patients could be kept in clinical remission after an operation. However, it is noteworthy that over time, a continuously increasing number of patients allocated to antireflux surgery, carried out at the discretion of the individual surgeon, were scored as treatment failures. A high proportion of surgical patients needed additional PPI treatment, and only 60% in those were kept in remission at 7 years. In the omeprazole arm, fewer than 50% remained in remission despite escalation of the drug dose over time.^10^

With the improved pharmacokinetics and bioavailability of the stereoisomer of omeprazole (esomeprazole), medical treatment today for GORD incorporates a more predictable and sustained level of acid inhibition.^11^ ^12^ The clinical implication of this is that larger proportions of patients can have symptoms controlled and the oesophagitis healed.^10^ ^13^ ^14^

In expert centres the laparoscopic approach to antireflux surgery predominates. The question therefore arises as to how laparoscopic Nissen fundoplication, carried out according to a standardised protocol in dedicated surgical centres, compares with updated medical treatment for GORD. We hereby present the 3-year efficacy results of a randomised and standardised long-term comparison of LARS with esomeprazole treatment in patients with chronic GORD.^16^

## METHODS

### Study design and objectives

The primary objective of this randomised open, parallel group, multicentre study was to compare the efficacy of long-term medical treatment with that of LARS in patients with chronic GORD, assessed through endoscopy, 24 h pH-metry and symptom response to esomeprazole. The participating centres had to be either academic units or affiliated to a University, and each operation had to be carried out or supervised in a standardised way^17^ by a consultant surgeon who specialised in this type of laparoscopic upper gastrointestinal (GI) surgery. Patients with a history of oesophageal, gastric or duodenal surgery, current or historical evidence of Zollinger–Ellison syndrome, primary oesophageal disorders (achalasia, schleroderma and primary oesophageal spasm), inflammatory bowel disease, dysplastic changes in a columnar-lined oesophagus or any condition associated with abnormal absorption from the GI tract were excluded from the study, as were patients with any other significant concomitant disease. Patients with potential for poor compliance were also excluded at the discretion of the investigator.

We applied a 3-month run-in period, which allowed baseline recordings and medical treatment with esomeprazole 40 mg once daily, to verify symptom response and healing of oesophagitis. After that, patients were randomised in blocks of four to either surgery or maintenance medical treatment with esomeprazole 20 mg once daily. The randomised design was selected to avoid bias in the selection of patients for medical or surgical treatment. All patients were eligible for either LARS or medical treatment, and their oesophagitis^18^ had to be no more than Los Angeles (LA) grade B at the time of randomisation, and GORD symptoms no more than mild. The local ethics committees approved the trial protocol, and written informed consent was obtained from all patients.

### Study schedule and measurements

The study schedule and principal measurements are summarised in detail in table 1. After Visit 1, when all patients had their baseline recording completed, they attended an “Investigational week”. If the patients had not taken PPIs during 7 days prior to Visit 1, they could start the investigational week immediately, which included endoscopy, biopsy sampling (oesophagus, Z-line, gastric antrum and corpus), laboratory screening and 24 h pH-metry with manometry and symptom association probability (SAP). *Helicobacter pylori* status was assessed in biopsy material from the antral and corpus parts of the stomach and, if clinically feasible, was to remain unchanged (ie, no eradication treatment) during the study period. This decision was taken because additional controlled data on the safety of profound and long-term acid inhibition on the morphology of the gastric mucosa seemed warranted, well aware of the current recommendations.^19^ In addition this design allowed us to evaluate further the eventual effect of *H pylori* infection on the subsequent clinical course.

**Table 1:** Study schedule and procedures

	Enrolment	Investigations	Week	Randomisation	Surgery	pH	Endoscopy	Endoscopy
Visit	1	2	3	4	5	7	8	12
Timing	−3 weeks	−6 weeks	−5 weeks	0	3 months	6 months	1 year	3 years
Endoscopy + biopsy	X		X	X			X	X
24 h pH-metry	X		X			X		
Surgery					X			
Symptom assessment*	X	X	X	X		X	X	X
Quality of life				X			X	
Adverse events*		X	X	X	X	X	X	X

*Also recorded at all intermediate 6 monthly visits after Visit 8.

All patients were treated with esomeprazole 40 mg once daily during the 3 month run-in period, but had to have been off PPIs for at least 7 days prior to the investigational endoscopy. If LA grade C/D oesophagitis was present at the investigational endoscopy, patients had a further endoscopic examination at Visit 4 following PPI treatment. If randomised to medical treatment, it started with esomeprazole 20 mg once daily, but the dose could be adjusted to 40 mg once daily after 8 weeks if symptoms were not controlled, and then to 20 mg twice a day for a further 8 weeks. If the patient was not controlled on this dose, this constituted treatment failure. If the dose was sufficient, one attempt was made to titrate the dose downwards but otherwise the patient remained on the higher dose.

Surgery had to be performed within 3 months of randomisation, using a laparoscopic approach, and consisted of a crural repair and the creation of a short floppy total fundoplication. Full details of the operative procedure and the perioperative/postoperative outcomes of this standardised procedure have been described elsewhere.^17^ Only surgical patients were required to attend Visits 5 (for surgery) and 6 (1 month postoperatively). Visit 7 took place 6 months after randomisation and, thereafter, clinic visits took place 6 monthly.

Follow-up endoscopy was planned at 1 and 3 years. At endoscopy, the oesophagus, cardiac region, stomach and duodenum were examined and biopsies were repeated. If there was any suspicion of Barrett’s oesophagus or malignancy, additional biopsies were taken.

Symptoms related to GORD were assessed at every visit (except after surgery). The investigator (in the case of LARS if possible a gastroenterologist and, after esomeprazole, a surgeon) asked standardised questions about heartburn, acid regurgitation and dysphagia severity. In addition, patients were asked about other GI symptoms including epigastric pain, flatulence, bloating, diarrhoea, ability to vomit and ability to belch. Quality of life (QoL) and patient-reported symptoms were assessed by administering the validated QOLRAD (quality of life in reflux and dyspepsia) and GSRS (gastrointestinal symptom rating scale) questionnaires^20^ ^21^ to patients at randomisation and annually thereafter. The translations of the questionnaires into different languages were done according to proposed guidelines and involved several independent translators.

During the follow-up period, patients in both treatment arms with moderate to severe recurrent GORD symptoms during at least three consecutive days were instructed to contact the clinic. They were then questioned about their treatment failure and offered an endoscopy (see following section).

Safety of treatments was assessed by comparing laboratory screening and histological parameters prerandomisation and after 1 and 3 years, and by recording serious adverse events (SAEs) throughout the study and those adverse events causing premature discontinuation.

### Treatment end points and statistical analyses

The main analyses were conducted using the intention to treat (ITT) population that included all randomised patients. A per protocol (PP) analysis was also performed on the primary efficacy data, and this included all randomised patients except those with major protocol violations. The safety population included all patients who received at least one dose of study drug and from whom postdose data were available.

The primary end point in this study was time to treatment failure, defined as follows for the two study treatments.

#### In the medical arm

The need for escalation in treatment for control of reflux disease was assessed at clinic visits by asking the question “Do you have sufficient control of your heartburn and acid regurgitation?” If the answer was no, and the patient stated a need for other regular medical treatment, the dose of esomeprazole was increased to 40 mg once daily for 8 weeks and could be adjusted to 20 mg twice a day for a further 8 weeks if symptoms had not resolved (24 h pH-metry and SAP were mandatory prior to a dose increase in esomeprazole-treated patients). If this proved insufficient to control symptoms, the patient was classified as a “treatment failure”.

#### In the surgical arm

The same questions were asked at clinic visits about symptom control in the surgical arm and if the answer was no and again backed up by the need for regular drug treatment (regular antisecretory drugs to control GORD symptoms, ie, ⩾4 weeks), the patient was classified as a “treatment failure”. The patient was also classified as a treatment failure if there were postoperative complaints requiring medical action, perioperative death, postoperative death within 30 days after surgery, dysphagia requiring further treatment, or any other requirement to reoperate for symptom control. In the case of functional oesophageal stenosis, one dilatation was allowed.

The outcome of the primary end point, time to treatment failure, is illustrated graphically by Kaplan–Meier remission curves in fig 2. These were compared statistically using the log rank test. In a post hoc analysis, mean scores of GI symptoms (none = 0, mild = 1, moderate = 2 and severe symptoms = 3) from 6 months to 3 years were compared using a two-sided, two sample t test. Change from the randomisation value to the average of the 1, 2 and 3 year values of the GSRS reflux dimension scores was compared using an analysis of variance (ANOVA), with values from the randomisation visit as covariate.

## RESULTS

### Study population

**Figure 1:**
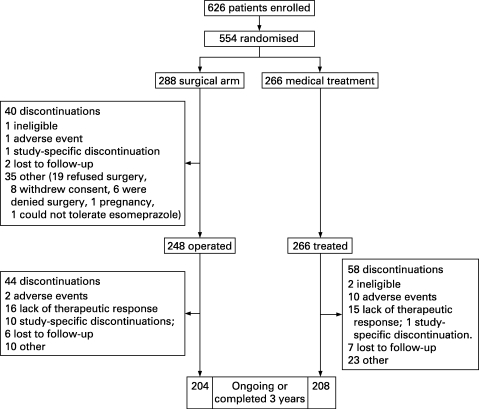
Patient flow during the 3 years from enrolment.

A total of 626 patients were enrolled for the study, of which 554 were randomised, 288 to surgery (40 of these were not operated on) and 266 to esomeprazole. The flow of patients through the study and reasons for withdrawal at each stage are summarised in fig 1. Of the 248 patients who had surgery, 204 have completed 3 years or are ongoing in the study and 44 have discontinued. In the medical arm, 208 of the 266 have completed 3 years or are ongoing and 58 have discontinued. The demographic details and GORD disease history for patients in each treatment group are presented in table 2. The two groups were well matched with regard to both their demographics and their history of GORD and current symptoms. The mean age was 45 years in both groups and the majority were male (69% and 75% for surgery and medical, respectively). Although around 30% in each group had a rather short history of verified reflux disease (<1 year), only 3% had a history of symptoms of less than 1 year and fewer than 4% of the patients presented with severe (LA grade C/D) oesophagitis. Around 40% in each group complained of moderate to severe heartburn, and around 30% of moderate to severe regurgitation at entry. Endoscopically suspected oesophageal metaplasia (defined as columnar metaplasia, whatever type, above the gastro-oesophageal junction) was diagnosed in 10.4% of surgical patients and in 9.4% of medical patients at entry. At baseline 14.3% of the patients in the medical group and 10.4% in those having LARS were infected with *H pylori*.

**Table 2:** Patient demographics and baseline characteristics

	Surgical arm	Medical arm
	n = 288	n = 266
Mean age (SD) years	44.8 (10.9)	45.4 (11.5)
% males	69.1	74.8
Mean BMI (SD)	27.2 (3.7)	27.3 (4.4)
Duration of verified reflux disease (%)		
<1 year	29.2	30.1
1–5 years	50.7	50.8
>5 years	19.4	18.8
LA grade of oesophagitis (%)		
No oesophagitis	46.5	48.5
Grade A	27.4	20.7
Grade B	22.2	27.1
Grade C	3.5	3.8
Grade D	0.3	0
Presence of Barrett’s oesophagus (%)	10.4	9.4
Heartburn severity (%)		
None	35.4	34.6
Mild	25.0	22.9
Moderate	24.3	24.4
Severe	15.3	18.0
Regurgitation severity (%)		
None	45.8	47.0
Mild	21.5	19.5
Moderate	24.3	24.8
Severe	8.3	8.6

BMI, body mass index; LA, Los Angeles.

### Treatment efficacy

**Figure 2:**
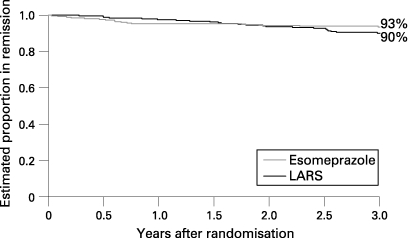
Proportion of patients in remission (ie, not classified as treatment failure). Intention to treat analysis after laparoscopic antireflux surgery (LARS) or on medical treatment.

**Figure 3:**
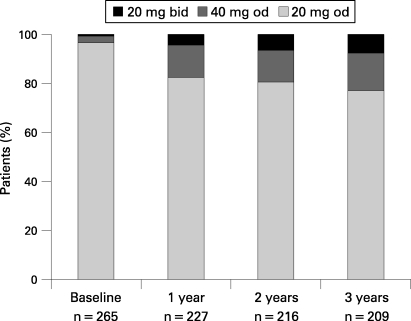
Bars represent the daily doses of esomeprazole used during the 3 years of the study. The numbers of patients at each time point are also given. bid, twice a day; od, once daily.

Time in remission (or time to treatment failure), the primary efficacy variable, is presented as Kaplan–Meier plots for the ITT population in fig 2. There was no significant difference between the treatments, with an estimated 90% of surgical patients remaining in remission compared with 93% of medically treated patients (p = 0.25), after 3 years. The results for the PP analysis were similar, with 90% for surgery and 95% for medical treatment (p = 0.045). At 3 years, 23% of the medical arm patients were on an increased dose of esomeprazole to control their symptoms, 8% on the maximum permitted dose schedule (fig 3).

#### GORD symptoms

**Figure 4:**
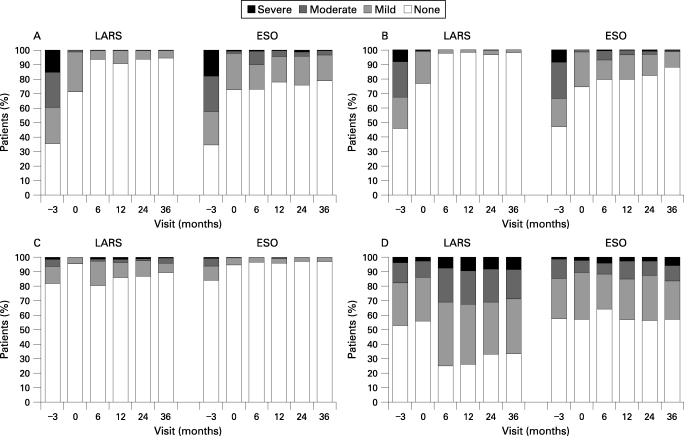
Symptoms of heartburn (A), acid regurgitation (B), dysphagia (C) and flatulence (D) in gastro-oesophageal reflux disease (GORD) patients randomised either to LARS (laparoscopic antireflux surgery) or daily esomeprazole (ESO).

The severity of heartburn and regurgitation reported by patients at each clinic visit throughout the study is illustrated in fig 4A and B, respectively. The medical group showed similar levels of heartburn and regurgitation at randomisation and all visits up to 3 years, while there was a decrease in both in the surgical group after randomisation. More patients reported heartburn in the medical group after randomisation (p<0.001); it tended to be mild and the number of reports was inversely related to the dose of esomeprazole. Hence, the numbers of symptomatic relapses were evenly distributed at 3 years (∼4% in each group).

Symptoms of dysphagia and flatulence are presented by severity in fig 4C and D, respectively. There was some dysphagia (mostly mild) after surgery, while very few medical patients had dysphagia (p<0.001). Flatulence was reported in both treatment groups, but more commonly after surgery (p<0.001).

*H pylori* infection had no impact on the clinical outcome in either of the groups (data not shown).

#### Quality of life

Low scores most often reported by GORD patients in the QOLRAD questionnaire are the food and drink dimension and the vitality dimension. Similarly, the reflux dimension of the GSRS questionnaire is highly sensitive. The mean scores for these dimensions at entry and following treatment are presented in table 3. Both the QOLRAD dimensions and the reflux dimension of GSRS showed greater improvement in the surgical group than in the medical group (p<0.001 for all dimensions).

**Table 3:** Mean dimensions scores for QOLRAD and GSRS at each visit

	Baseline	1 year	2 years	3 years
Surgery				
QOLRAD*				
Vitality	6.28 (1.08)	6.84 (0.52)	6.87 (0.46)	6.90 (0.31)
Food and drink	6.16 (1.16)	6.78 (0.6)	6.83 (0.49)	6.85 (0.4)
GSRS†				
Reflux	1.81 (1.07)	1.18 (0.44)	1.21 (0.51)	1.18 (0.42)
Medical				
QOLRAD*				
Vitality	6.21 (1.22)	6.42 (0.92)	6.45 (0.93)	6.53 (0.85)
Food and drink	6.19 (1.12)	6.34 (0.96)	6.35 (0.95)	6.38 (0.91)
GSRS†				
Reflux	1.73 (1.03)	1.66 (0.88)	1.66 (0.96)	1.63 (0.88)

*7 = no problems, 6 = minimal problems.

†1 = no discomfort, 2 = minimal discomfort.

Treatment comparisons and p values are described in the text.

GSRS, gastrointestinal symptom rating scale; QOLRAD, quality of life in reflux and dyspepsia.

### Safety

SAEs of any type (including postoperative complications) were reported by 21% of surgical patients and by 14.3% of medical patients. Ten percent of the non-operated patients and 3% of the non-randomised patients also reported SAEs. During the study, there was one death of a 68-year-old man in the medical arm, due to pneumonia. There were two reports of myocardial infarction, one in each treatment arm. Adverse events led to study discontinuation in 0.8% of the surgical patients and in 3.8% of the medical patients. There was no perioperative mortality and only 3% morbidity within the hospital stay or during the 30 days after surgery. The most common SAEs are summarised, by system organ class, in table 4.

**Table 4:** Number of patients with serious adverse events (SAEs; by system organ class) for SAEs occurring in >1% of any treatment group

Number of patients with SAEs	Refused surgery n = 40	Surgery n = 248	Medical n = 266	Not randomised n = 72
	n (%)	n (%)	n (%)	n (%)
Injury, poisoning, procedural	1 (2.5)	15 (6.0)	2 (0.8)	1 (1.5)
Gastrointestinal disorders	0	12 (4.8)	5 (1.9)	1 (1.5)
Musculoskeletal/connective tissue	0	2 (0.8)	8 (3.0)	0
Infections and infestations	1 (2.5)	3 (1.2)	6 (2.3)	0
General disorders	0	5 (2.0)	4 (1.5)	0
Cardiac disorders	1 (2.5)	4 (1.6)	3 (1.1)	0
Neoplasms, benign/malignant	0	2 (0.8)	6 (2.3)	0
Reproductive system including breast	0	1 (0.4)	4 (1.5)	0
Respiratory, thoracic, mediastinal	0	5 (2.0)	1 (0.4)	0
Vascular disorders	0	3 (1.2)	3 (1.1)	0
Hepatobiliary disorders	1 (2.5)	3 (1.2)	0	0

## DISCUSSION

This study demonstrates that long-term (3 year) laparoscopic Nissen fundoplication and esomeprazole medication are similarly effective treatments for GORD, based on symptom evaluation, endoscopy and overall QoL measures. There were differences between the groups in relation to the outcome of other GI symptoms, severity of persistent reflux symptoms and overall vitality. In the surgical arm, the level of reflux symptom control, as also reflected in some QOLRAD dimensions, was somewhat better than on long-term medical treatment. This was, however, counterbalanced in the surgical group by those patients who suffered slightly more GI symptoms of a postfundoplication nature, such as abdominal pain and other functional symptoms. Postfundoplication problems are often regarded as a major stumbling block in the surgical approach to treating GORD but, within this trial, their frequency and degree were minimal, despite well-structured follow-up specifically designed to detect such problems. The success of both surgical and medical treatment arms, with 90% and 93% complete remission of symptoms, respectively (based on a strict definition of treatment failure), indicates that both treatments were highly effective.^22^^–^26

Our outcome data show dysphagia rates that differ only slightly after surgery compared with medical treatment. This may come as a surprise to some observers. Similarly, there was little difference in symptoms of epigastric pain and bloating, which to some extent was unexpected.^17^ ^26^^–^29 Also the complication rates after the initial operation, at 3%, were exceptionally low.^28^^–^30 This high standard of surgery was achieved in 40 centres across Europe, so the results are applicable to any centre that can show itself to be sufficiently experienced and suitably trained in the technique of LARS.

The outcome variables, as currently assessed, suggest that improvements have been made in the long-term management of GORD relevant to both medical and surgical treatments. A similar trial comparing open ARS with the first generation of PPIs^10^ showed a lower degree of improvement in “like for like” symptom analyses. However, despite the similarities in design and outcome assessments, corresponding comparisons should be interpreted with caution. The current surgical outcomes when compared with previous studies could be due to a number of factors. First, we believe that the standardisation of the surgical technique within this trial is unique and not only contributes to the improved outcomes, but also shows that the actual operative techniques and details can be disseminated widely. As a consequence of this standardisation, all hiatal hernias were fully reduced and a crural repair was achieved to retain this anatomical correction.^27^ A second factor that may have contributed to the quality of the surgical results is the selection of centres with high volume and surgeons with established experience. Within each country such teams should be able to provide this quality in routine clinical practice. Thirdly, the laparoscopic technique may not only be superior to previous open techniques,^28^ ^31^ ^32^ but may also lend itself to critical review by the whole team, who can view (and record) the operation in all of its detail.

When considering medical treatment for GORD, there is a rationale for reliable and regular acid control for greater consistency in therapeutic response across the GORD population. The improved levels of acid inhibition and minimal side effects associated with the use of esomeprazole, irrespective of the severity of the GORD,^11^^–^15 most probably contributed to the outcome in the present study.

Both treatments were well tolerated and there were no safety concerns after 3 years of follow-up. Surgery has become safer with the introduction of laparoscopy, minimising complication risks (3% in this trial)^32^ and reducing the need for^28^ ^29^ ^31^ repair of subsequent incisional hernia (1% seen in this study so far). This study does not give any clear indication of the 5–10 year outcomes, but other reports indicate that the operation will be as durable as its open equivalent.^32^

In conclusion, this study has shown that in GORD patients, selected for inclusion in this trial, good quality surgical or medical treatment achieves very high standards of patient outcome. Both treatments were highly effective, safe and well tolerated.
